# Supplementary material for: Comprehensive Annotation and Functional Exploration of MicroRNAs in Lettuce
Source: Front Plant Sci. 2021 Dec 24;12:781836. doi: 10.3389/fpls.2021.781836 (PMC8739914; doi:10.3389/fpls.2021.781836)
Supplement: Supplementary file 2 [file Data_Sheet_8.ZIP › GSM2230750.plot/Lsa-miR396b_Lsat_1_v5_gn_5_72841.1_2120_TPlot.pdf]

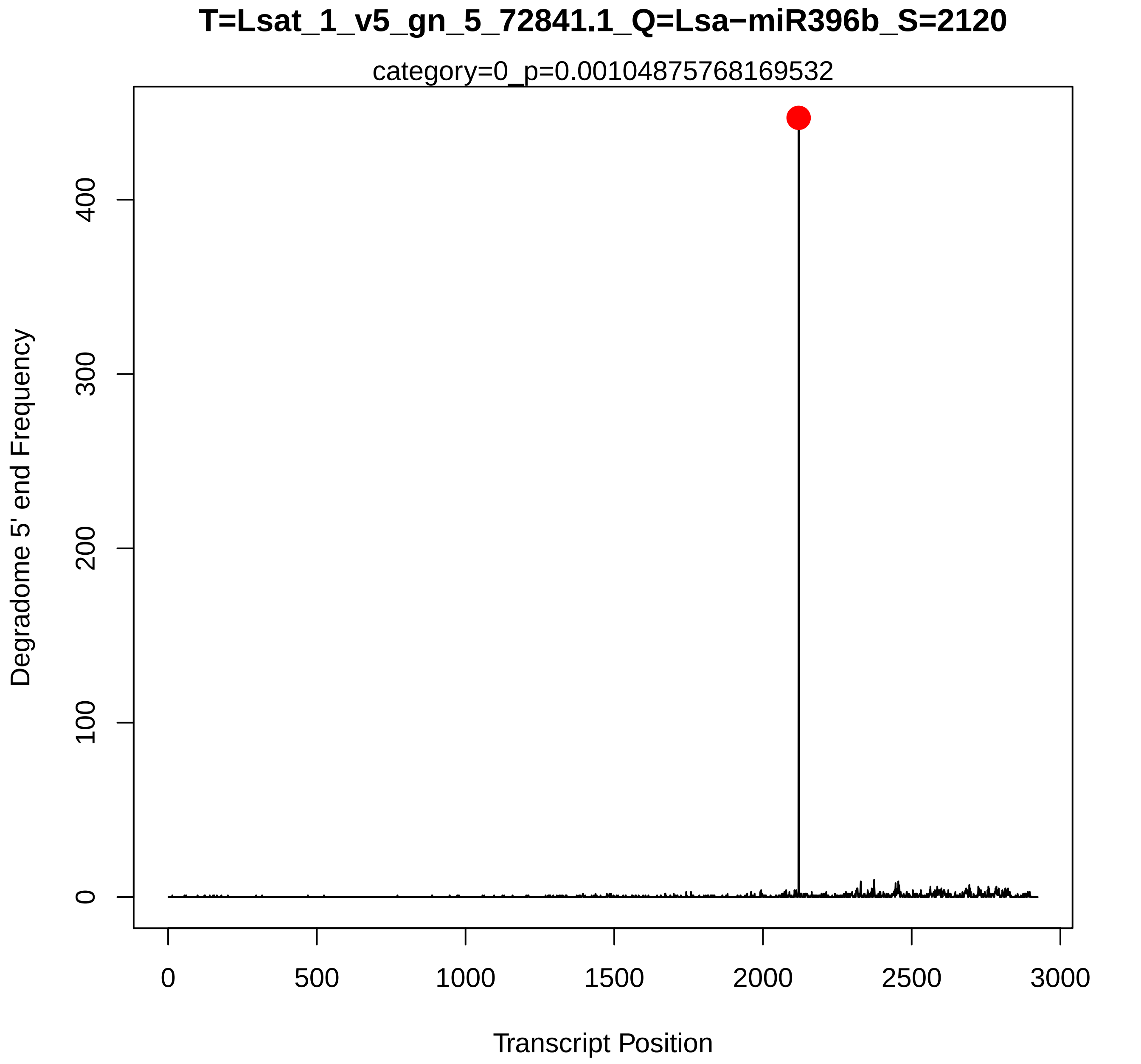
T=Lsat_1_v5_gn_5_72841.1_Q=Lsa−miR396b_S=2120
category=0_p=0.00104875768169532
Degradome 5' end Frequency
0
100
200
300
400
Transcript Position
0
500
1000
1500
2000
2500
3000
